# Modern work patterns of “classic” versus millennial family doctors and their effect on workforce planning for community-based primary care: a cross-sectional survey

**DOI:** 10.1186/s12960-020-00508-5

**Published:** 2020-09-21

**Authors:** Lindsay Hedden, Setareh Banihosseini, Nardia Strydom, Rita McCracken

**Affiliations:** 1 Faculty of Health Sciences, Simon Fraser University, Blusson Hall, Room 11300, 8888 University Drive, Burnaby, BC V5A 1S6 Canada; 2 British Columbia Academic Health Science Network, Vancouver, Canada; 3 Department of Family Medicine, Providence Health Care, Vancouver, Canada; 4 Department of Family Practice, Faculty of Medicine, University of British Columbia, Vancouver, Canada; 5 Department of Family and Community Medicine, Vancouver Coastal Health, Vancouver, Canada

## Abstract

**Background:**

There are ongoing accessibility challenges in primary care in British Columbia, Canada, with 17% of the population not having a regular source of care. Anecdotal evidence suggests that physicians are moving away from a community-based comprehensive practice model, which could contribute to shortages. Thus, we aimed to identify and describe how family physicians are currently organizing their primary care practices in a large health region in British Columbia and to examine differences between newer graduates and more established physicians.

**Methods:**

Data for this cross-sectional study were drawn from an annual physician privileging survey. *N* = 1017 physicians were invited to participate. We categorized practice style into five distinct groupings and compared features across respondent groups, including personal and practice location characteristics, hospital and teaching work, payment and appointment characteristics, and scope of practice. We discuss the implications of styles of practice and associated characteristics on health workforce policy and planning.

**Results:**

We received responses from 525 (51.6%) physicians. Of these, 355 (67.6%) reported doing at least some community-based primary care. However, only 112 (21.3%) provided this care full time. Most respondents supplemented community-based work with part-time hours in focused practice, hospitals, or inpatient facilities. We found diversity in the scope and style of practice across practice models. Compared to established physicians, new graduates (in practice less than 10 years) work more weekly hours (more patient care, and paperwork in particular). However, we found no difference between new and established physicians in the odds of providing any or full-time community-based primary care.

**Conclusions:**

Despite a lack of formalized structural reform in British Columbia’s primary care system, most physicians are finding alternative ways to model their practice and shifting away from work at single-location, community-based clinics. This shift challenges assumptions that are relied on for workplace planning that is intended to ensure adequate access to longitudinal, community-based family medicine.

## Background

Approximately one in six British Columbians do not have a regular family doctor [1], matching the Canadian national average. News reports of patients looking for a family physician and being unable to find one are ubiquitous, and most physician practices are not currently accepting new patients [2–6]. The gap between demand and supply exists despite a consistent increase in numbers of family physicians per capita since 1986 [7, 8], and stable per-physician remuneration [9, 10]. This suggests that either underlying changes in practice are reducing supply or demand is increasing.

Since federal Medicare legislation was passed in 1966, there have been no substantive structural changes to how primary care is delivered in British Columbia (BC), and it is assumed that the vast majority of physicians are practicing in the “classic” model. We define this as working in a community-based physician-owned and physician-operated practice, either alone or with a small group of physician colleagues, providing comprehensive full-scope care to a large panel of patients under a fee-for-service (FFS) model [11]. Non-physician providers such as registered nurses, nurse practitioners, or other health professionals are not part of classic practices and have not been integrated into the primary care system more broadly.

There are signals, however, that the classic primary care model may no longer reflect how the majority of primary care physicians are practicing, and therefore that measures of supply within the system could be substantially inflated [12]. This could at least partly explain the conflicting observation of both increasing demand and increasing supply. Activity levels and patient panel sizes have declined substantially over time [10]. Fewer physicians are managing inpatient care for their own patients, or are otherwise providing services at non-office-based locations [13, 14], and comprehensiveness of care appears to be declining [14, 15].

Furthermore, there are options available for physicians who do not wish to run a solo or shared private community-based practice, such as hospitalist positions or locum tenens [16–18], and these roles are becoming more available. Physicians may choose to work in part-time “focused practice” type roles (e.g., sports medicine, palliative care, addiction treatment) [19].

There is also a small but growing body of evidence that suggests that new physicians are structuring their practices differently than more established physicians and that they may be more likely to choose some of these non-classic roles. For example, only 61% of family medicine residents in Western Canada stated that they intended to provide comprehensive care, and a full 28% report that they will focus their practice in specific clinical areas only [19]. Newer physicians provide a different basket of services than their more established counterparts [15, 20]. They are also more likely to prefer non-FFS remuneration and to choose a model that does not involve clinic ownership and management [21, 22].

If physicians working in blended models, clinically focused practice, or full-time hospitalist or locum positions account for a substantial percentage of overall supply of primary care physicians, and particularly if these models are more common among newer-to-practice physicians, there will be fewer physicians remaining to provide longitudinal, comprehensive, community-based primary care now and increasingly so in future. This could help to explain the co-occurring observations of increasing supply with no change in unmet need, but we are unaware of any studies that have examined this either in BC or elsewhere in Canada. The objectives of this cross-sectional survey analysis were to identify and describe current models of primary care practice in a Health Authority in BC and to examine whether and in what ways newer graduates model their practices differently than more established physicians.

## Methods

### Setting and context

Primary care in BC, Canada, is publicly funded and privately delivered. Primary care physicians, who deliver the vast majority of care, have traditionally worked in solo or small-group physician-owned and physician-operated practices. They are paid by the provincial health insurance plan on an FFS basis. Physicians pay overhead and staffing costs from gross FFS billings. There is a limited role for interdisciplinary teams or non-physician providers such as registered nurses and nurse practitioners. This is beginning to shift as the Ministry of Health implements some structural reforms (though these occurred after the data for this study were collected) [23–25].

BC is divided into five health regions. The largest region by population, Vancouver Coastal Health (VCH), provides services to 1.25 million British Columbians (25% of the provincial population) across 12 municipalities and four regional districts. It operates a network of hospitals and acute care facilities, specialized community health centers, residential care facilities, and home support services.

Data for this cross-sectional study were drawn from information that is routinely collected as part of VCH’s annual privileging process, which physicians are required to complete to confirm their eligibility to provide any services at a VCH facility. The research team designed a survey to address the annual privileging and workforce planning needs within VCH and to elicit physician perspectives on their models of practice. The survey was reviewed and approved by the Regional Medical Director of Primary Care for VCH.

The survey captures information on how physicians are currently structuring their practices, and asks additional questions that support health authority-wide human resource planning such as planned retirements. The data were linked with publicly available demographic and training information from the College of Physicians and Surgeons of BC, including gender, year of graduation, and training location. While the privileging process is required, the survey component is optional.

Physicians who had clinical privileges with VCH in 2018 were invited (via email) to participate in the survey. Participation reminders were sent 1 and 5 weeks following the initial invitation. Responses were collected between January 30 and April 15, 2018. Six weeks after the initial invitation, physicians were also invited to have their data removed if they had reconsidered since first answering the survey. The survey was delivered online using REDCap. Researchers were presented with de-identified data for analysis.

### Survey content and variables

We asked respondents to select between four options that describe their main model of primary care practice: all or some community-based primary care (CBPC), hospital or inpatient facility only, locum only, or non-clinical (provide no patient care). We grouped those respondents who indicated that they provide at least some CBPC based on the number of self-reported hours per week spent working in locations where CBPC is provided (solo, two to four, or five + physician clinics in the community that a patient can access without a referral). The three groups used were full-time CBPC (> 37.5 h per week), mostly CBPC plus some other work (20–37.5 h per week CBPC), and mostly other work plus some CBPC (< 20 h per week CBPC). We also asked that they indicate whether their main model of practice was focused, general, or mixed (general but also with a specific clinical focus), and asked about the provision of specific services and care to special populations.

The survey also included a core set of demographic and work questions, including hours worked per “typical” week (broken into specific tasks areas such as patient care, paperwork, and business activities), number of practice locations, retirement intentions, and responsibilities for call coverage.

### Statistical analyses

We excluded respondents who reported that they do not provide any patient care or who did not respond to core questions about model of practice. To determine the representativeness of our sample, we compared demographic (year of graduation and gender) and training (location) characteristics for survey respondents with the total population of primary care physicians practicing within BC area, using *χ*^2^ tests. These data are presented in Supplement 2. Data for this comparison were drawn from the College of Physicians and Surgeons of BC public listing.

For descriptive analysis, we used one-way ANOVA and *χ*^2^ tests to compare demographic and practice characteristics across core models of practice (some/all CBPC, hospital/facility only, or locum) and among physicians who provide at least some CBPC (less than half time, more than half time, or full time). We used the same test to compare demographic and practice characteristics between new graduates (physicians in practice less than 10 years) and more established physicians.

To assess the adjusted effect of demographic and practice characteristics on model of practice, we used a two-stage modeling approach. We used a multivariable logistic model to assess which variables were associated with providing at least some CBPC. Then among the subsample of physicians who did report that they provide at least some CBPC, we constructed a second logistic model to assess the effect of the same set of demographic and practice characteristics on whether or not they provide CBPC care full time.

Finally, we conducted a series of *χ*^2^ tests looking for differences in provision of specific clinical services across full time, greater than half time, and less than half time spent providing CBPC. Services included maternity care, substance abuse treatments, non-office-based care, serving special populations, and the use of technology to support accessible patient care. All analyses were conducted in Stata IC/15.1.

## Results

**Fig. 1:**
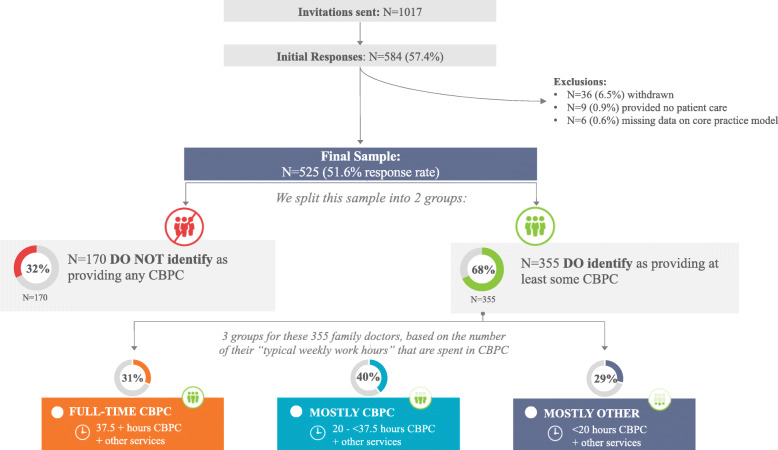
Study flow diagram

In total, 1017 physicians were initially invited to participate in the study. Of these, 584 physicians completed the survey (57.4%; Fig. 1); however, 38 (6.5%) of them subsequently requested that their data be withdrawn. An additional nine (0.9%) responses were removed for physicians reporting that they do not provide any patient care, and six (0.6%) were discarded because of incomplete responses to core practice model questions, leaving a final sample of 525 (51.6%). Of the respondents in that final sample, 56% (291) are women, 21.3% (112) trained outside of Canada, and 21.1% (111) provide at least some care in a rural practice location.

On average, respondents had been in practice for 22.5 years (SD = 13.3), and 131 (25.0%) intend to retire within the next 5 years. Respondents work an average of 44.1 h per week (SD = 15.2), and 30 of those hours (SD = 13.1) were dedicated to direct patient care.

Among respondents, 355 (67.6%) reported doing at least some community-based primary care: 112 (31.6%) provide these services 37.5 h per week or more, 141 (39.7%) between 20 and 37.5 h per week, and 102 (28.7%) less than 20 h per week (Table 1). On average, physicians who reported that their model of practice includes at least some CBPC spent 27 h per week (SD = 16.0) on that work and 18.9 h per week (SD = 15.7) on other work. Of respondents, 42 (11.8%) provide no services in CBPC clinics, despite selecting this as their core model of practice. Among the remaining 170 who reported not providing CBPC, 97 (18.5%) report working entirely in a hospital or inpatient facility and 73 (13.9%) are locums.

**Table 1:** Study sample and characteristics of models of practice

Population/service	Full-time CBPC	Mostly CBPC	Mostly other work	Total ***N*** = 355	Test statistic (*χ*^**2**^)
	***N*** = 112 (31.6%)	***N*** = 141 (39.7%)	***N*** = 102 (28.7%)		
*Maternity services*					
Pre-natal to 24 weeks	76 (67.8)	97 (68.8)	44 (43.1)	217 (61.1)	19.5^‡^
Prenatal after 24 weeks	20 (17.9)	35 (24.8)	18 (17.7)	73 (20.6)	2.6
Deliveries	10 (8.9)	22 (15.6)	13 (12.8)	45 (12.7)	2.5
Postpartum care	62 (55.4)	68 (48.2)	31 (30.4)	161 (45.4)	14.2*
*Provides services to special populations*					
Elders with frailty	44 (39.3)	39 (27.7)	24 (23.5)	107 (30.1)	7.0*
LGBT	14 (12.5)	28 (19.9)	16 (15.7)	58 (16.3)	2.5
Transgender	11 (9.8)	23 (16.3)	12 (11.8)	46 (13.0)	2.5
Refugees	12 (10.7)	15 (10.6)	11 (10.8)	38 (10.7)	0.0
Individuals with chronic pain	28 (25.0)	32 (22.7)	18 (17.7)	78 (22.0)	1.8
Adults with disabilities	16 (14.3)	16 (11.4)	6 (5.9)	38 (10.7)	4.0
Palliative care	25 (22.3)	30 (21.3)	22 (21.6)	77 (21.7)	0.0
People with HIV	13 (11.6)	25 (17.7)	19 (18.6)	57 (16.1)	2.4
People with substance use disorders	20 (17.9)	43 (30.5)	38 (37.3)	101 (28.5)	10.3^†^
People with mental health conditions	36 (32.1)	51 (26.2)	31 (30.4)	118 (33.2)	1.0
*Substance use treatment*					
Treats substance use disorders	41 (36.6)	75 (53.2)	55 (57.9)	171 (49.1)	10.9^†^
Prescribes methadone	23 (20.5)	48 (34.0)	47 (49.5)	118 (33.9)	19.2^‡^
Prescribes naloxone	31 (27.7)	58 (41.3)	45 (47.4)	134 (38.5)	9.1*

*p < 0.05

^†^*p* < 0.01

^‡^*p* < 0.0001

At the bivariate level, we observed significant differences among practice models with respect to gender distribution, average years in practice, training location, rural practice location, work hours, and number of practice locations. We observed no difference in provision of call coverage or retirement planning (Table 1). Among the three groups of respondents who provided at least some CPBC, we observed significant bivariate differences in patient panel size; in focused, mixed, or general practice; and in working in at least one hospital/facility or emergency room. More physicians who provide CBPC for less than 20 h per week reported working in clinically focused practice compared with those who work between 20 and 37.5 h per week or more than 37.5 h per week (20.2%, compared to 4.3% and 1.8% respectively). More physicians who work less than 20 h per week also had small patient panel sizes, but work at more locations than those who spend more time providing CBPC.

We found some differences in populations and services across the CBPC groups (Tables 2 and 3). More physicians providing CBPC full time reported providing prenatal and postpartum care than either part-time group. More physicians in this group also reported providing house calls, hospital visits, and residential care, and caring for elders with frailty. A significantly larger percentage of physicians who reported spending less than 20 h per week working in CBPC provide services for individuals with substance use disorders, including treating those disorders and prescribing methadone and buprenorphine/naloxone.

**Table 2:** Special services and populations (among physicians providing any community-based primary care, *N* = 355)

Characteristic, *N* (%)	Full-time CBPC *N* = 112 (21.3%)	Mostly CBPC *N* = 141 (26.9%)	Mostly other work *N* = 102 (19.4%)	Full-time hospital/ facility *N* = 97 (18.5%)	Locum *N* = 73 (13.9%)	Total *N* = 525	Test statistic
*Demographics*							
Gender (women)	42 (37.5)	92 (65.3)	60 (58.8)	53 (54.6)	44 (60.3)	291 (55.5)	*χ*^2^ = 20.6^‡^
Ave. years in practice (SD)	26.2 (13.0)	21.1 (11.9)	21.3 (13.6)	25.0 (12.8)	17.7 (15.0)	22.5 (13.3)	*F* = 6.2^†^
First 10 years in practice	27 (24.1)	42 (29.8)	40 (39.2)	26 (14.8)	40 (22.7)	175 (33.3)	*χ*^2^ = 23.7^‡^
International medical graduates	39 (34.8)	36 (25.5)	18 (17.7)	13 (13.4)	6 (8.2)	112 (21.3)	*χ*^2^ = 25.6^‡^
Any rural practice	16 (14.3)	36 (25.5)	15 (14.7)	15 (15.5)	29 (39.7)	111 (21.1)	*χ*^2^ = 24.3^‡^
*Work hours (total)*^2^	54.3 (12.7)	44.9 (12.0)	39.1 (14.1)	39.5 (15.2)	40.0 (18.0)	44.1 (15.2)	*F* = 22.1^‡^
Patient care	38.3 (11.0)	31.1 (10.8)	24.3 (12.7)	26.4 (12.9)	27.8 (14.5)	30.0 (13.1)	*F* = 21.3^‡^
Paperwork	10.8 (6.0)	9.3 (6.1)	6.3 (4.2)	7.4 (6.5)	8.3 (5.9)	8.6 (6.0)	*F* = 9.2^‡^
Business activities	2.7 (2.9)	2.2 (2.5)	1.6 (2.2)	1.4 (1.7)	1.6 (2.1)	2.0 (2.4)	*F* = 5.6^†^
Clinical supervision	2.4 (4.9)	2.0 (3.0)	2.2 (5.6)	3.7 (6.0)	.4 (1.1)	2.2 (4.6)	*F* = 5.5^†^
Other^3^	3.1 (6.8)	3.3 (5.2)	6.6 (10.5)	5.3 (7.5)	3.8 (11.7)	4.3 (8.1)	*F* = 2.3
*Ave. number of practice locations (SD)*	1.7 (1.0)	2.1 (1.1)	2.6 (1.3)	2.0 (1.4)	5.8 (4.0)	2.6 (2.3)	*F* = 62.3^‡^
Works in 1+ hospital ER	3 (2.7)	14 (10.0)	22 (21.6)			39 (11.0)	*χ*^2^ = 19.8^‡^
Works in 1+ inpatient facility	24 (21.4)	44 (31.2)	47 (46.1)			115 (32.4)	*χ*^2^ = 15.0^†^
*Practice type*^*4*^							
General	63 (56.3)	56 (39.7)	14 (13.7)			133 (38.3)	*χ*^2^ = 54.2^‡^
Focused	2 (1.8)	6 (4.3)	19 (20.2)			27 (7.8)	
Mixed	47 (42.0)	79 (56.0)	61 (59.8)			187 (53.9)	
*Panel size (across all locations)*^*5*^							
≤ 100	8 (7.1)	18 (12.8)	44 (43.1)			70 (20.1)	*χ*^2^ = 104.1^‡^
101–500	8 (7.1)	33 (23.4)	27 (26.5)			68 (19.5)	
501–1000	23 (20.5)	33 (23.4)	9 (8.8)			65 (18.7)	
1001–2000	45 (40.2)	47 (33.3)	11 (10.8)			103 (29.6)	
> 2000	28 (25.0)	10 (7.1)	4 (3.9)			42 (12.1)	
*Provides call coverage*	94 (83.9)	114 (80.6)	79 (77.5)	79 (81.4)	51 (69.9)	417 (79.4)	*χ*^2^ = 6.1
*Planning to retire within 5 years*	27 (24.1)	34 (24.1)	20 (19.6)	29 (29.9)	21 (28.8)	131 (25.0)	*χ*^2^ = 3.5

*p < 0.05

^†^*p* < 0.01

^‡^*p* < 0.0001

^1^Missing *N* = 27

^2^Total hours were asked independently of the work categories below. The categories therefore do not always sum to the total

^3^Includes classroom teaching, leadership, committees, research, and others

^4^*N* = 355. Only asked for those physicians providing full-time CBPC, mostly CBPC, or mostly other, and excluding hospital/facility and locums

^5^Missing *N* = 7

**Table 3:** Off-site care and technology for patient appointments (among physicians providing any community-based primary care, *N* = 355)

Population/service	Full-time CBPC	Mostly CBPC	Mostly other work	Total ***N*** = 355	Test statistic
	***N*** = 112 (31.6%)	***N*** = 141 (39.7%)	***N*** = 102 (28.7%)		
*Offers any off-site visits*					
House calls	80 (71.4)	96 (68.1)	47 (46.1)	223 (62.8)	17.5^‡^
Hospital visits	79 (70.5)	89 (63.1)	48 (47.1)	216 (60.9)	12.9^†^
Residential care visits	56 (50.0)	69 (48.9)	24 (23.5)	149 (42)	20.0^‡^
Outreach services	12 (10.7)	25 (17.7)	14 (13.7)	51 (14.4)	2.5
Care in schools	4 (3.6)	3 (2.1)	4 (3.9)	11 (3.1)	0.8
Correctional facilities	1 (0.9)	1 (0.7)	2 (2.0)	4 (1.1)	0.9
Other	2 (1.8)	4 (2.8)	5 (4.9)	11 (3.1)	1.8
*Technology for patient care*					
Email	49 (43.8)	47 (33.3)	28 (27.5)	124 (34.9)	6.5*
Phone	107 (95.5)	134 (95.0)	84 (82.4)	325 (91.6)	15.7^‡^
Text message	28 (25.0)	28 (19.9)	14 (13.7)	70 (19.7)	4.3
Skype/videoconference	28 (25.0)	10 (7.0)	6 (6.4)	70 (19.7)	0.9

*p < 0.05

^†^*p* < 0.01

^‡^*p* < 0.0001

New graduates work more weekly hours (more patient care, and paperwork in particular) than established physicians. They also work in more locations (Table 4). We also found differences between new and established physicians in terms of model of practice, with more new graduates working in locum practice and fewer full-time or mostly in CPBC. New graduates are also more likely to work in mixed practice and less likely to work in focused or general practice models.

**Table 4:** Comparison between new and established physicians

Characteristic, *N* (column %)	New graduate *N* = 175 (33.3%)	Established *N* = 350 (66.7%)	Total *N* = 525	Test statistic
*Demographics*				
Gender (women)	108 (61.7)	183 (52.4)	291 (55.5)	*χ*^2^ = 4.06*
International medical graduates	27 (15.4)	85 (24.3)	112 (21.3)	*χ*^2^ = 5.45*
Any rural practice	129 (73.7)	285 (81.4)	414 (78.9)	*χ*^2^ = 4.16*
*Work hours (total)*^*1*^	46.2 (15.0)	43.0 (15.2)	44.1 (15.2)	*F* = 5.11*
Patient care	31.2 (12.8)	29.4 (13.2)	30.0 (13.1)	*F* = 2.24
Paperwork	8.9 (5.5)	8.4 (6.3)	8.6 (6.0)	*F* = 0.91
Business activities	1.9 (2.3)	2.0 (2.4)	2.0 (2.4)	*F* = 0.03
Clinical supervision	2.3 (4.5)	2.2 (4.7)	2.2 (4.6)	*F* = 0.00
Other^2^	3.9 (8.9)	4.6 (7.7)	4.3 (8.1)	*F* = 0.72
*Practice model*				*χ*^2^ = 23.67^‡^
Full-time CBPC	27 (15.4)	85 (24.3)	112 (21.3)	
Mostly CBPC	42 (24.0)	99 (28.3)	141 (26.9)	
Mostly other	40 (22.9)	62 (17.1)	102 (19.4)	
Hospital/facility	26 (14.9)	71 (20.3)	97 (18.5)	
Locum	40 (22.9)	33 (9.4)	73 (13.9)	
*Ave. number of practice locations (SD)*^*3*^	2.6 (1.2)	1.9 (1.1)	2.1 (1.2)	*F* = 46.6^‡^
Works in 1+ hospital ER	16 (14.7)	23 (9.35)	39 (11.0)	*χ*^2^ = 2.19
Works in 1+ inpatient facility	50 (45.9)	65 (26.4)	115 (32.4)	*χ*^2^ = 13.05^‡^
Works in 1+ walk-in clinic	31 (11.8)	29 (11.8)	60 (16.9)	*χ*^2^ = 9.27^†^
*Practice type*^*3*^				*χ*^2^ = 5.18
General	65 (61.3)	122 (50.6)	187 (53.9)	
Focused	4 (3.8)	23 (9.5)	27 (7.8)	
Mixed	37 (34.9)	96 (39.8)	133 (38.3)	
*Provides call coverage*	147 (84.0)	270 (77.1)	417 (79.4)	*χ*^2^ = 3.36

*p < 0.05

^†^*p* < 0.01

^‡^*p* < 0.0001

^1^Total hours were asked independently of the work categories below. The categories therefore do not always sum to the total.

^2^Includes classroom teaching, leadership, committees, research, and others

^3^*N* = 355. Only asked for those physicians providing full-time CBPC, mostly CBPC, or mostly other, and excluding hospital/facility and locums

Our multivariable analyses found that physicians who trained internationally had significantly higher odds of reporting that some or all of their work is CBPC (some CBPC: OR 2.92, 95% CI 1.70–5.01; full-time CPBC: OR 2.03, 95% CI 1.20–3.43) than physicians who trained in Canada (Table 5). Women had significantly lower odds of providing full-time CBPC (OR 0.37, 95% CI 0.23–0.59). There was no significant difference in the odds of providing any or full-time CBPC between new or established family physicians once we adjusted for the effects of gender, training location, and practice location.

**Table 5:** Multivariate modeling results—predictors of models of primary care practice

Characteristic	Model 1: Any CBPC	Model 2: Full-time CBPC*
	Odds ratio (95% CI)	Odds ratio (95% CI)
New graduate (< 10 years since graduation)	0.79 (0.53, 1.17)	0.72 (0.42,1.22)
Gender (women)	0.94 (0.64, 1.37)	**0.37 (0.23, 0.59)**
Training (international)	**2.92 (1.70, 5.01)**	**2.03 (1.20, 3.43)**
Practice location (rural)	**0.61 (0.39, 0.96)**	0.52 (0.27, 1.00)

*Locums, hospital/facility-based physicians excluded

## Discussion

We found that while two thirds of our respondents reported providing at least some CBPC, only 21% of the sample provide that care full time. This substantial move away from the classic general practitioner model suggests that capacity for longitudinal CBPC is much more limited than headcount metrics or billings-based full-time equivalents would suggest. At the same time, only 5% of our sample are working less than 20 h per week, suggesting that those who are spending less than 37.5 h per week doing CBPC are working full-time hours but spending time in non-CBPC locations such as focused practices, inpatient facilities, or emergency rooms (ERs). Furthermore, new graduates work on average 3.5 h per week more than established physicians, in contrast to the commonly heard narrative of millennial family doctors working part time and being a cause for erosion our primary care capacity [26].

This study is one of the few available descriptions of modern work patterns by family physicians in Canada. Existing literature relies heavily on billings data, which has not allowed for description of practice across multiple locations, fails to distinguish between community-based and other venues for delivering primary care, and cannot be used to calculate hours spent working. Even full-time equivalent calculations based on dollars billed, such as those produced by CIHI [27], do not account for whether the work a physician is doing is CBPC, hospitalist work, locum work, or something else. It is also one of the few studies that examine differences in work models and patterns between new graduates and established physicians. While it supports the existing finding that substantial numbers of new graduates do not (or do not intend to) provide comprehensive primary care [28, 29], it extends that work by providing direct comparison with more established physicians.

Our results also suggest that primary care physicians are finding alternative ways to model their practices, shifting away from single-location community-based clinics, despite the lack of formalized structural reform in BC’s primary care system. Assessments of whether these new work patterns are aligned with patient need and what is driving physicians to move away from CBPC are beyond the scope of this paper, but both are critical areas of study that should be undertaken in the future. At a minimum, however, the fact that there is a substantial ongoing population need for increased access to longitudinal CBPC suggests that greater attention needs to be paid to what jobs are being offered to family physicians and how they compare to the models of practice physicians are creating for themselves.

Other ways to address the unmet need for primary care may involve a move to an interdisciplinary team-based model of care and formally incorporating registered nurses, nurse practitioners, and other non-physician providers into the primary care system. The BC Ministry of Health has recently started to implement a substantial suite of reforms that includes a core focus on interdisciplinary teams [23–25], organized into Patient Medical Homes [30]. The Patient Medical Homes will still be physician-owned and physician-operated, and FFS remuneration is expected to be the dominant form of remuneration. While in theory this could reduce the number of physicians required to meet population health needs, the extent of the uptake of these voluntary reforms among new graduates and established physicians remains to be seen, as will the extent to which the reforms can successfully reduce the level of unmet need.

### Limitations

This study used a cross-sectional survey-based analysis and has the standard set of limitations associated with that approach. Our respondents differ from the broader population of primary care physicians within VCH and BC. For example, women are over-represented in our sample (55.4%) compared to all primary care physicians practicing within VCH (48.4%) and across BC more broadly (42.0%; Supplement 1). Our sample also had a smaller percentage of physicians who trained outside of Canada (21.8%) compared to the larger BC physician population (32.6%), but similar to the percentage for VCH (25.7%).

Given that women were over-represented in our sample, and the relationship we observed between gender and the odds of doing any or full-time CBPC, it is possible we are underestimating the proportion of physicians working in either of these models at least somewhat. Furthermore, because this survey was collected as part of annual privileging, it is likely we are overestimating the proportion of family physicians who work solely or in part in a hospital or facility. Hours spent providing CPBC are likely to be underestimated in a rural population, where primary clinics may be nested within or closely affiliated with a hospital. However, because VCH is a primarily urban health authority, we expect any effect on our results to be minimal.

### Conclusions

This cross-sectional survey supports the growing body of evidence on access and capacity challenges in community-based primary care in British Columbia. Models of practice are moving away from full-time care in the community and toward models that blend community-based care with work in other practice locations. Approaches to workforce planning in the context of primary care need to account for shifting models of practice in their estimates of current and future primary care capacity. Furthermore, ongoing reforms to primary care, including the introduction of team-based Patient Medical Homes and Primary Care Networks, should be made with the intention of making community-based practice an attractive and viable option relative to hospitalist, locum, and blended practice models.

## Supplementary information

**Additional file 1.** Representativeness of study sample. Table S1.1: Comparison between study sample and all primary care physicians practicing within Vancouver Coastal Health. Table S1.2: Comparison between study sample and all primary care physicians practicing within British Columbia.

## Data Availability

The data that support the findings of this study are available from Providence Health Care, but restrictions apply to availability. Data were used under license for the current study and so are not publicly available. Data are however available from the authors upon reasonable request and with permission of Providence Health Care.

## Abbreviations

ANOVA: Analysis of variance
BC: British Columbia
CBPC: Community-based primary care
ER: Emergency room
FFS: Fee-for-service
VCH: Vancouver Coastal Health
