# Supplementary material for: Modern work patterns of “classic” versus millennial family doctors and their effect on workforce planning for community-based primary care: a cross-sectional survey
Source: Hum Resour Health. 2020 Sep 21;18:67. doi: 10.1186/s12960-020-00508-5 (PMC7504652; doi:10.1186/s12960-020-00508-5)
Supplement: Supplementary file 1 — Additional file 1. Representativeness of study sample. Table S1.1: Comparison between study sample and all primary care physicians practicing within Vancouver Coastal Health. Table S1.2: Comparison between study sample and all primary care physicians practicing within British Columbia. [file 12960_2020_508_MOESM1_ESM.docx]

**Supplement 1: Representativeness of study sample**

Table S1.1: Comparison between study sample and all primary care physicians practicing within Vancouver Coastal Health

|  | **Study sample**  **N=525** | **Total VCH primary care physicians** | **Total**  **N=1928** | **Test statistic (X^2^)** |
| --- | --- | --- | --- | --- |
|  |  | **N=1460** |  |  |
| Gender |  |  |  | X^2^= 7.6^†^ |
| Men | 234 (44.6) | 753 (51.6) | 987 (49.7) |  |
| Women | 291 (55.4) | 707 (48.4) | 998 (50.3) |  |
| Training |  |  |  | X^2^= 3.6 |
| Within Canada | 412 (78.5) | 1085 (74.3) | 1497 (75.4) |  |
| International | 113 (21.5) | 375 (25.7) | 488 (24.6) |  |

*p<0.05 ^†^p<0.01 ^‡^p<0.0001

Table S1.2: Comparison between study sample and all primary care physicians practicing within British Columbia

|  | **Study sample**  **N=525** | **Total BC primary care physicians** | **Total**  **N=6460** | **Test statistic (X^2^)** |
| --- | --- | --- | --- | --- |
|  |  | **N=6935** |  |  |
| Gender |  |  |  | X^2^= 35.3^‡^ |
| Men | 234 (44.6) | 3441 (58.0) | 3675 (56.9) |  |
| Women | 291 (55.4) | 2492 (42.0) | 2785 (43.1) |  |
| Training |  |  |  | X^2^= 27.5^‡^ |
| Within Canada | 412 (78.5) | 3998 (67.4) | 4410 (68.3) |  |
| International | 113 (21.5) | 1937 (32.6) | 2050 (31.7) |  |

*p<0.05 ^†^p<0.01 ^‡^p<0.0001
